# Increasing quality of life in pulmonary arterial hypertension: is there a role for nutrition?

**DOI:** 10.1007/s10741-018-9717-9

**Published:** 2018-06-16

**Authors:** Paulien Vinke, Suzanne M. Jansen, Renger F. Witkamp, Klaske van Norren

**Affiliations:** 1 Nutrition and Pharmacology Group, Division of Human Nutrition and Health, Wageningen University, Stippeneng 4, 6708 WE Wageningen, The Netherlands; 2 Actelion Pharmaceuticals Nederland B.V., Woerden, the Netherlands

**Keywords:** Pulmonary arterial hypertension, Nutrition, Deficiencies, Exercise, Lifestyle, Review

## Abstract

Pulmonary arterial hypertension (PAH) is a progressive disease primarily affecting the pulmonary vasculature and heart. PAH patients suffer from exercise intolerance and fatigue, negatively affecting their quality of life. This review summarizes current insights in the pathophysiological mechanisms underlying PAH. It zooms in on the potential involvement of nutritional status and micronutrient deficiencies on PAH exercise intolerance and fatigue, also summarizing the potential benefits of exercise and nutritional interventions. Pubmed/Medline, Scopus, and Web of Science were searched for publications on pathophysiological mechanisms of PAH negatively affecting physical activity potential and nutritional status, and for potential effects of interventions involving exercise or nutritional measures known to improve exercise intolerance. Pathophysiological processes that contribute to exercise intolerance and impaired quality of life of PAH patients include right ventricular dysfunction, inflammation, skeletal muscle alterations, and dysfunctional energy metabolism. PAH-related nutritional deficiencies and metabolic alterations have been linked to fatigue, exercise intolerance, and endothelial dysfunction. Available evidence suggests that exercise interventions can be effective in PAH patients to improve exercise tolerance and decrease fatigue. By contrast, knowledge on the prevalence of micronutrient deficiencies and the possible effects of nutritional interventions in PAH patients is limited. Although data on nutritional status and micronutrient deficiencies in PAH are scarce, the available knowledge, including that from adjacent fields, suggests that nutritional intervention to correct deficiencies and metabolic alterations may contribute to a reduction of disease burden.

## Introduction

Pulmonary arterial hypertension (PAH) is a progressive disease affecting the arteries of the lungs. It is defined by a mean pulmonary artery pressure ≥ 25 mmHg at rest, an end-expiratory pulmonary artery wedge pressure (PAWP) ≤ 15 mmHg, and a pulmonary vascular resistance > 3 Wood units. There are different etiologies suggested for PAH, as the disease can (1) develop idiopathically (without a known cause); (2) be heritable (e.g., result from inherited mutations in genes coding for specific proteins like bone morphogenic protein receptor 2); (3) be associated with certain diseases like connective tissue disease, congenital heart disease, portal hypertension, or HIV; or (4) be induced by drugs or toxins [1]. The prevalence of all types of PAH in Europe is estimated to be in the range of 15–100 cases per million population. Prognosis of PAH is poor, with 1- and 3-year survival rates of 87 and 67%, respectively [1, 2]. The guidelines for the diagnosis and treatment of pulmonary hypertension from the European Society of Cardiology (ESC) and the European Respiratory Society (ERS) describe several risk stratification criteria for determination of prognosis of PAH patients (1-year mortality) [1]. Predictors of poor prognosis include advanced functional class, poor exercise capacity, and right ventricular dysfunction [1]. In heart failure (CHF) patients, right ventricular dysfunction and exercise intolerance are known to contribute to muscle wasting [3]. Many of the clinical characteristics of PAH such as heart failure, exercise intolerance, functional class, muscle wasting, and comorbidities are strongly associated with each other. These characteristics have also been taken along in the REVEAL score, which has been developed to predict disease progression and survival of PAH patients using prognostic criteria [4].

Malnutrition and muscle wasting are known clinical manifestations of cachexia, which is often seen in PAH as well [5]. Cachexia as such also contributes to a higher mortality risk and poorer quality of life [3, 6]. At the same time, there appears to be hardly any knowledge on the relevance of nutritional status in PAH. For those readers less familiar with some of the terminology used, Box 1 provides some formal definitions of cachexia, sarcopenia, and nutritional and micronutrient deficiency [7]. Interestingly, in case of CHF, which to some degree shows overlapping pathology with PAH, nutritional supplementation with fish oil, rich in n-3 fatty acids, has shown to improve disease progression. In addition, increased protein intake has proven to be effective in attenuating muscle wasting in CHF patients [8]. Specifically in PAH patients, iron deficiency is known to have a negative impact on exercise tolerance and fatigue [1, 9]. With this in mind, we hypothesize that nutrition impacts disease progression and quality of life in case of PAH as well. Furthermore, we envisage an opportunity to improve predictive modeling and quality of life for PAH by including nutritional status as a factor. Given the clinical manifestations of PAH, it is expected that effects resulting from optimizing nutritional status of PAH patients will at least include improved maintenance of muscle mass and function and ultimately quality of life.

**Box 1:** Formal definitions of used nutritional terminology

Box 1 Formal definitions Cachexia—loss of lean tissue mass, involving a weight loss greater than 5% of body weight in 12 months or less in the presence of chronic illness or as a body mass index (BMI) lower than 20 kg/m^2^. According to the definition of Evans et al., three of the following five criteria are also required: decreased muscle strength, fatigue, anorexia, low fat-free mass index, an increase of inflammation markers, anemia, or low serum albumin [7]. Sarcopenia—loss of muscle mass and function, especially muscle strength and gait speed, commonly associated with aging [7] Nutritional deficiency—an inadequate supply of essential nutrients from the diet, potentially resulting in malnutrition and disease Micronutrient deficiency—an inadequate presence of vitamins or minerals in the body, potentially leading to symptoms or increasing general disease risk Nutritional status—the condition of the body with respect to factors influenced by the diet: the levels of nutrients in the body and the ability to maintain normal metabolic function	

The pathophysiology of PAH involves endothelial dysfunction in the pulmonary vasculature, metabolic shifts in vascular cells, hypertrophy and proliferation of smooth muscle cells, and unprogrammed growth of neointimal, medial, and adventitial layers, leading to thickening and occlusion of the small- and medium-sized pulmonary arteries [10]. Three known pathways involved in PAH are currently used as therapeutic targets. These are the endothelin pathway, the nitric oxide pathway, and the prostacyclin pathway [11]. Current therapies consist of endothelin receptor antagonists (ERAs), phosphodiesterase 5 (PDE5) inhibitors, a soluble guanylate cyclase (sGC) agonist, prostacyclin, prostacyclin analogues, and a selective IP receptor agonist [1]. These compounds aim to reduce pulmonary vascular resistance, provide relief of symptoms, enhance exercise capacity, and delay disease progression, but do not cure the disease once it has started. Therefore, additional treatment options for this incurable disease are clearly needed. Among these, treating muscle dysfunction, enhancing physical activity potential, and decreasing fatigue could be of great importance. Exercise programs, psycho-social support, and dietary consultation are offered to PAH patients depending on local availability, insurance coverage, and laws and regulations. However, there is little information about nutritional status or daily activity of PAH patients, or about possible strategies to increase the quality of life of patients by means of nutritional or lifestyle interventions. The ESC/ERS Guidelines for the diagnosis and treatment of pulmonary hypertension [1] contain very limited nutritional and lifestyle recommendations. Therefore, this review aims to gather the information on nutritional status, physical performance, and activity levels of PAH patients. In addition, clinical outcomes of previously investigated nutrition and lifestyle interventions are discussed.

Evidence-based nutrition and exercise interventions for PAH are limited, but established lifestyle interventions for CHF might be useful for PAH patients as well based on the similarities in pathophysiology. The 2016 Guidelines of the European Society of Cardiology (ESC) recommend regular aerobic exercise training in patients with heart failure and stable heart failure with reduced ejection fraction to improve functional capacity and reduce the risk of hospitalization (class I, level A recommendation) [12]. Nutritional advice for heart failure includes avoiding excessive salt intake (> 6 g/day) and fluid restriction in case of severe heart failure [8, 12]. Supplementation of omega-3 polyunsaturated fatty acids (N3 PUFA) has shown beneficial effects on mortality and hospitalization in patients with heart failure. Furthermore, high-caloric, protein-rich nutritional supplements or branched-chain amino acids can be beneficial in case of muscle wasting or cachexia [8]. Further research is needed to investigate whether these recommendations are also beneficial for PAH patients.

## Methods

We used Pubmed/Medline, Scopus, and Web of Science to search for publications connecting the pathophysiological mechanisms of PAH to impaired physical activity, exercise intolerance, chronic fatigue, and nutritional deficiencies often observed in patients with PAH. We also searched for nutritional and exercise interventions to prevent or modulate these symptoms. For the literature search, we used specific combinations of the following search terms: pulmonary arterial hypertension, PAH, pathophysiology, symptoms, mechanism, right ventricular failure, skeletal muscle alterations, inflammation, hypoxia, dysfunctional energy metabolism, lipid accumulation, insulin resistance, satiety hormones, nutritional status, nutrition, deficiency, vitamin, mineral, protein, lipid, fatty acid, carbohydrate, iron, magnesium, calcium, vitamin D, omega 3 fatty acids, omega-3 PUFA, docosahexaenoic acid (DHA), eicasopentaenoic acid (EPA), alpha-linolenic acid (ALA), activity, daily activity, lifestyle, exercise tolerance, exercise capacity, exercise intervention, and quality of life.

## Activity level and physical performance of PAH patients

A limited number of studies available underline the prevailing clinical idea that activity levels of patients decline with disease progression [2, 13, 14]. A study using accelerometry to estimate activity levels in 23 PAH and chronic thromboembolic pulmonary hypertension (CTEPH) patients found a relationship between activity and disease severity. Severely impaired patients (mean pulmonary arterial pressure (mPAP) 50 ± 7 mmHg) were inactive for longer periods during the night, and were less active during the day than modestly impaired patients (mPAP 33 ± 7 mmHg) [13]. Long nocturnal rest and reduced activity during the day were also reported to correlate with an overall poorer prognosis [13]. Another study showed that PAH patients spent significantly more time in sedentary activities than matched healthy controls and that this impacted all levels of physical activity [14]. A more recent paper of Alami et al. supports these findings, reporting that patients with PAH experience such an increase in symptoms like shortness of breath and fatigue that they have increased problems performing normal daily tasks such as body care, household chores, parenting, and leisure activities during progression of the disease [2]. There are currently more studies underway using activity tracking, such as the wearable wrist device Actigraph, which enables better recording of the activity pattern of PAH patients and the impact of treatment on activity levels (e.g., the TRACE study, clinicaltrials.gov identifier NCT03078907).

## Nutritional status of PAH patients

PAH eventually leads to right heart failure, which is found to contribute to loss of skeletal muscle mass [3, 6]. Loss of skeletal muscle is a strong predictor of death in (left) heart failure patients [15]. Next, loss of skeletal muscle mass is often accompanied with loss of weight and fat mass [3, 6]. Muscle loss in general, diagnosed as sarcopenia or sometimes as cachexia, is related to a worsened prognosis and lower quality of life of chronically ill patients [8, 16]. The Society on Sarcopenia, Cachexia and Wasting Disorders (SCWD) defines cachexia as “a loss of lean tissue mass, involving a weight loss greater than 5% of body weight in 12 months or less in the presence of chronic illness or as a body mass index (BMI) lower than 20 kg/m2.” Often, three of the following five criteria are also required: decreased muscle strength, fatigue, anorexia, low fat-free mass index, an increase of inflammation markers, anemia, or low serum albumin [7]. Sarcopenia focuses on muscle loss and is defined as “loss of muscle mass and function, especially muscle strength and gait speed, associated with aging” [7] (see also Box 1). Improving nutritional status during the earliest stages of chronic diseases such as heart failure is an important factor in reducing the rate at which muscle wasting develops. Despite this, only few studies have investigated nutritional status in PAH.

In 2015, Kawamoto showed a close relationship between PAH with inferior vena cava (IVC) dilatation, poor nutritional status, and low BMI in a small prospective study with 8 PAH patients [3]. An older, cross-sectional study with 20 heart failure patients showed that these patients have an 18% higher resting metabolic rate and risk to develop sarcopenia than healthy age-matched controls [17]. Based on the similarities in pathophysiology, it could be hypothesized that PAH patients also have an increase in energy expenditure, although future studies will have to confirm this. Furthermore, drugs used to treat PAH, specifically epoprostenol, prostacyclin analogues, and other drugs that target the prostacyclin pathway, are known to induce side effects that may impact nutritional status and exercise tolerance: nausea, loss of appetite, vomiting, diarrhea, jaw pain, musculoskeletal pain, and fatigue [2].

## Disease-related characteristics influencing daily activity or nutritional status

### Right ventricular failure

In PAH patients, the pulmonary vascular system is obstructed and less elastic, which leads to an increased afterload of the right ventricle with decreased right ventricular cardiac output [18–20]. This, in turn, decreases the left ventricular preload which leads to a decreased left ventricular output. The decreased left ventricular output causes a reduced oxygen supply to the muscles both at rest and during exercise, contributing to exercise intolerance [21–25]. In addition to a reduced cardiac output, it has been suggested that PAH is associated with impaired chronotropic capacity [18, 26], which is associated with downregulation of β-adrenoreceptor activity in the right ventricle [27]. These effects prevent adequate adaptation of cardiac output and systemic blood pressure during exercise, which can further contribute to exercise intolerance. The pathological changes in the heart and other organs and their contribution to exercise intolerance are well described elsewhere [28], and include cardiac factors (right ventricular function), skeletal muscle dysfunction (decreased oxidative enzymes, reduced muscle fiber size), pulmonary factors (endothelial dysfunction, arterial stiffness), and other factors (e.g., inflammation and oxidative stress). Based on this, it is clear that right ventricular failure plays an important role in the exercise intolerance often found in PAH patients and that the responsible pathophysiological changes are relatively well studied.

### Skeletal muscle alterations

Multiple causes of reduced daily activity of PAH patients have been proposed. In addition to shortness of breath and fatigue, skeletal and respiratory muscle alterations have also been described. These include a switch from type I fiber to type II fiber in skeletal muscle, increased muscle protein degradation, a reduced muscle capillary density, lower aerobic enzyme activity, mitochondrial abnormalities, and impaired calcium homeostasis [29–32]. The cross-sectional area and force-generating capacity of muscle cells from the left ventricle have been reported to be reduced by 30 and 25%, respectively [33]. Furthermore, forearm muscle weakness was found to correlate with respiratory muscle strength and exercise capacity, independent of hemodynamic severity in a prospective study with 24 idiopathic PAH (IPAH) patients (66% female) [34]. Pathological changes and reduced strength in the diaphragm muscle of PAH patients or in animal models of PH have been found by several authors [35–37]. The changes in the diaphragm muscle might be different from those in skeletal muscle. Instead of a type I to type II fiber switch as seen in skeletal muscle, a switch from type II fiber to type I fiber was observed in the diaphragm of animal models of heart failure [38, 39]. Alterations in the skeletal and respiratory muscles contribute to exercise intolerance in PAH patients. Whether changes in fiber type in skeletal and respiratory muscles in models of PAH are different (as was found in animal models of heart failure) remains to be studied. There is also a lack of studies into the potential effect of lifestyle interventions such as nutrition or exercise on skeletal muscle alterations in PAH.

### Inflammation

Altered immune function and chronic inflammation are increasingly recognized features of PAH [10, 40–42]. In PAH patients, an accumulation of perivascular inflammatory cells including mast cells, macrophages, dendritic cells, and T and B lymphocytes has been found in the pulmonary vasculature and lung tissue [43]. In addition, chronically elevated serum levels of the proinflammatory cytokines IL-1b, IL-6, IL-8, IL-10, MCP-1, fractalkine, CCL5/RANTES, and TNF-α and chemokines have been reported [19, 42, 43]. Inflammatory conditions such as connective tissue diseases are associated with increased incidence of PAH, indicating that inflammation might play a significant role in the development and progression of PAH [19, 42]. This is supported by findings that in PAH patients, inflammation is associated with worse clinical outcome [43, 44].

Inflammatory cytokines such as IL-6 and TNF-α are known to impact nutritional status and to induce muscle wasting in other chronic diseases [45, 46]. Inflammation induces muscle protein breakdown via multiple mechanisms, such as via increased protein degradation through the ubiquitin-proteasome system (UPS), mitochondrial dysfunction, and autophagy [45–47]. The role of inflammation on mitochondrial dysfunction in chronic heart failure seems twofold: IL-6 has been shown to prevent mitochondrial dysfunction in cardiomyocytes, whereas TNF-α induces mitochondrial dysfunction in the same cell type [48]. It has been found that TNF-α worsens endothelial function in CHF, causing a decreased blood supply to other organs such as the skeletal muscle and the gut. As a consequence, less nutrients are absorbed from the gut and transported to other organs. At the same time, IL-6 induces the acute phase inflammatory response, which requires essential amino acids. Skeletal muscle is an important source of essential amino acids when delivery via the food is limited. Therefore, more skeletal muscle is broken down [47]. TNF-α, IL-6, and other inflammatory cytokines induce transcription of atrogenes, such as MuRF1 and MAFbx/Atrogin-1, via FOXO transcription factors. The E3-ubiquitin ligases MuRF-1 and MAFbx/Atrogin-1 are the main ligases in skeletal muscle that identify proteins for removal via the UPS. The UPS gets activated, which leads to increased protein degradation [45]. Proinflammatory cytokines such as TNF-α and IL-1 also inhibit food intake via an anorexigenic effect in the brain [49].

Based on this information, it can be hypothesized that inflammation decreases the nutritional status of PAH patients and that it can induce muscle wasting or cachexia. This, in turn, supposedly augments the response to inflammation. In this way, a vicious cycle can develop that can only be interrupted when both inflammation and nutritional status are targeted at the same time [50–52]. Further research is needed to investigate to what extent alterations in immune function and inflammatory processes observed in PAH could affect nutritional status and whether nutritional supplementation might alter the inflammatory process in PAH.

### Dysfunctional cellular energy metabolism

Oxygen is important for mitochondrial ATP production via oxidative phosphorylation. In PAH patients, abnormalities in energy production, heme synthesis, and mitochondrial function have been hypothesized to result from chronic hypoxia [9, 53].

A factor important for these impairments is hypoxia-inducible factor 1α (HIF-1α): a hypoxia-sensitive transcription factor. Under normoxic circumstances, HIF-1α is very unstable and rapidly degraded. Under chronic hypoxic conditions, as occurring in PAH patients, HIF-1α stabilizes and moves to the nucleus to induce expression of HIF target genes [54]. Expression of these genes promotes the delivery of oxygen to tissues and maintenance of ATP levels [55]. However, chronic activation of HIF also induces proteins that alter energy metabolism and cell proliferation and cause vascular remodeling, resulting in the development of pulmonary arterial remodeling and pulmonary hypertension [54]. HIF-1α activates glycolytic genes, increasing the production of lactate from pyruvate, and inactivates enzymes necessary for mitochondrial oxidative phosphorylation [56]. This process is known as aerobic glycolysis and often leads to an energy deficit [56, 57]. An increase in lactate levels might even suppress food intake, because lactate has an appetite-inhibiting effect [58]. The production of high amounts of lactate could also contribute to dysfunction of the right ventricle [59]. Due to the metabolic shift from oxidative to glycolytic metabolism, the mitochondrial membrane gets hyperpolarized, leading to proliferation of pulmonary artery smooth muscle cells (PASMC) and reduced apoptosis. This process may contribute to the development of pulmonary hypertension [56].

Other factors that could contribute to mitochondrial dysfunction in PAH are deficiencies in caveolin-1 and mutations in BMPRII. Mutated BMPRII shifts glucose metabolism of pulmonary arterial endothelial cells (PAECs) to anaerobic glycolysis, causing decreased ATP production, mitochondrial DNA damage, and apoptosis of endothelial cells [56]. A deficiency in caveolin-1 leads to activation of endothelial nitric oxide synthase (eNOS) and the development of ROS, also contributing to impaired mitochondrial functioning [56].

Both hypoxia and a decrease in cellular ATP can induce muscle breakdown and dysfunction which further reduces the exercise capacity of the patient [29].

In summary, due to chronic hypoxia present in PAH, there are changes in mitochondrial energy metabolism that lead to inefficient ATP production in the cells. Lactate, which occurs as a side product of aerobic glycolysis, might contribute to right ventricular dysfunction and potentially leads to a reduced dietary intake, thereby negatively influencing the clinical status of the patient. However, future studies have to test this hypothesis. A lower cellular ATP production might also induce muscle breakdown and dysfunction itself, although more studies to support this hypothesis are needed.

### Lipid accumulation

In the healthy human heart, the major source of energy (60–90%) is provided by fatty acid oxidation (FAO). The other 10–40% mainly comes from glucose oxidation [59]. In PAH patients, due to the Warburg effect, glucose utilization in the heart increases, while FAO is reduced. Despite this, there is an increase in uptake of fatty acids by the fatty acid (FA) transporter CD36 [60, 61]. Lipid accumulation in cardiomyocytes and skeletal muscle cells is therefore a common feature in PAH patients [62]. Cytosolic accumulation of FA leads to a more abundant production of long-chain acyl-CoA in the cytoplasm, which can be converted into intracellular lipid intermediates, such as triglycerides, diacylglycerol, and ceramides, causing cardiac lipotoxicity. These intermediates induce insulin resistance due to multiple mechanisms, such as impairing pyruvate dehydrogenase (PDH), inhibiting insulin receptor substrate 1 (IRS-1) and glucose transporter type 4 (GLUT4) expression [60]. Next to insulin resistance, the lipid intermediates are also related to the development of cardiac dysfunction and right heart failure [60]. Insulin resistance is related to skeletal muscle weakness in CHF patients, although it is unclear whether this relationship is causal [63].

Cardiac dysfunction and right heart failure lead to fatigue and hypoxia, both of which contribute to exercise intolerance. This altogether leads to muscle protein breakdown. The combination of immobility and the inability to use fat can lead to a sarcopenic obesity phenotype (high BMI, low muscle mass), a malnourished phenotype that is also more and more encountered in cancer [64] and is associated with increased morbidity and mortality in this disease. Based on the above-described mechanisms and cause-effect relationships between lipid intermediates, insulin resistance, and skeletal muscle weakness, the prevalence of sarcopenic obesity in PAH patients needs further investigation.

### Insulin resistance

A higher prevalence of insulin resistance has been reported in PAH patients than in the general population, and this may be a disease modifier or risk factor for PAH [65–68]. Insulin resistance is known to be a risk factor for cardiovascular disease (CVD) in general. It has been linked to diseases that share pathophysiological characteristics with PAH such as idiopathic cardiomyopathy or congestive heart failure [65]. Inflammatory cytokines, seen in the pathogenesis of PAH, are likely to contribute to insulin resistance [69]. Obesity in PAH patients may contribute to the development of insulin resistance if coupled with low daily activity levels [65], but insulin resistance has been found independently of obesity in PAH patients as well [66]. In general, insulin resistance induces energy deficit in the muscle, which triggers muscle protein breakdown. From this, it becomes clear that there are many observed associations between insulin resistance and PAH, but little is known about whether it is a symptom of, or a contributing factor to, the disease. More research is needed to show whether insulin or glucose levels play a role in the pathophysiology of PAH and whether it contributes to a loss of muscle mass in PAH patients.

### Gut-derived satiety hormones

Deregulations in the expression of satiety hormones, such as increased levels of the anorexigenic hormone PYY after a meal, have been found in a small study with nine cardiac cachectic patients with primary PAH [5]. In addition to its systemic effects, PYY reduces the digestion rate by decreasing GI motility, gall bladder emptying, and gastric emptying, which further contributes to a reduction in food intake [70]. Larger trials should show whether an increase in the anorexigenic hormone PYY or changes in other gut-derived satiety hormones after a meal contribute to reduced food intake in PAH patients.

## Disease-related nutritional deficiencies and intervention trials investigating their possible connection to quality of life

### Iron deficiency

Iron deficiency is common in patients with idiopathic and heritable forms of PAH. The prevalence has been estimated to be between 30 and 65%, dependent on the type of PAH and status of the patient [1, 9, 71, 72]. Iron deficiency is defined by reduced serum iron (normal = 9.0–30.0 μmol/L), serum ferritin (normal = 3–400 μg/L (males), 30–150 μg/L (females)), and transferrin saturation (normal = 16–45%) [73]. In patients with IPAH, a low iron status has been related to a reduced exercise capacity, measured as the 6-min walk test, and to poorer survival [9, 74]. Iron deficiency was reported to be independent of the presence of anemia [9, 72, 75] and not associated with a worsened right ventricle function [76].

Availability of iron influences the pulmonary vasoconstrictor response to hypoxia and the basal pulmonary artery pressure [77, 78]. Iron status influences modulation of the pulmonary circulation, but also myocardial and skeletal muscle function. Iron is essential for oxygen transport and functions as a cofactor in several mitochondrial oxidative enzymes and the respiratory chain. Inhibition of dietary iron uptake by the negative regulator of plasma iron levels, hepcidin, might form a mechanism behind the iron deficiency. The main iron transporter of the intestine (ferroportin) is inhibited by hepcidin, resulting in a decreased intestinal absorption of iron. Hepcidin levels are higher in patients with PAH. Moreover, a low intestinal absorption of iron is supported by reports that the response to oral iron supplementation is low in PAH patients [71, 74]. High hepcidin levels might be due to dysfunctional BMPRII signaling and/or the presence of chronic inflammation. Normally, high hepatic iron levels induce bone morphogenic protein 6 (BMP6) expression, which stimulates hepcidin transcription via BMP response elements 1 and 2 (BMP-RE1 and BMP-RE2) on the hepcidin gene. Inflammatory cytokines such as IL-1beta and IL-6 stimulate hepcidin transcription via different mechanisms, leading to excessive hepcidin production. IL-1beta might even provide the onset signal, as it induces the transcription of IL-6. This excessive hepcidin production also happens when there are no high hepatic iron levels present and thereby leads to inflammation-induced anemia [79].

In summary, iron deficiency is common in PAH patients and contributes to the disease itself and to exercise intolerance by influencing the pulmonary circulation, myocardial and skeletal muscle function, and oxidative energy metabolism. High levels of hepcidin, induced by inflammatory cytokines and/or dysfunctional BMPRII signaling, lead to inhibition of dietary iron uptake.

### Iron supplementation

The response to oral iron supplementation in PAH patients is low, which is likely due to inhibition of iron uptake by hepcidin in the digestive tract [71, 74]. Therefore, more recent studies investigated the effect of intravenous infusion of iron in the form of ferric carboxymaltose. For example, Viethen et al. and Ruiter et al. studied the effect of a single dose of 1000 mg ferric carboxymaltose. They used observation periods of 8 and 12 weeks in 20 and 15 PAH patients, respectively [73, 74]. Both studies found improvement of serum iron status and quality of life, as measured by the patient-reported Short Form 36 (SF-36) questionnaire, and showed minimal side effects. The study of Viethen et al. also found an improvement in the 6-min walk distance (6MWD), whereas Ruiter and colleagues did not, although they did find improved endurance exercise capacity and mitochondrial oxidative capacity [73, 74]. A larger study into the effects of parenteral iron replacement in at least 60 patients with IPAH is underway (The “Supplementation of Iron in Pulmonary Hypertension” (SIPHON) Phase II clinical trial) [80]. More randomized controlled trials are needed to show whether parenteral iron replacement is more successful in treating iron deficiency than oral iron supplementation and if this leads to a better quality of life of PAH patients.

### Vitamin D

Vitamin D deficiency is linked to musculoskeletal, metabolic, and cardiopulmonary diseases and to disorders of the immune system [81, 82]. It has been suggested that vitamin D influences smooth muscle cell proliferation and endothelial function, which are both affected in PAH [81, 83, 84]. Pulmonary hypertension was associated with vitamin D_3_ deficiency in a group of 40 systemic sclerosis patients [84]. In a small prospective uncontrolled longitudinal study, weekly supplementation of 50,000 IU cholecalciferol (vitamin D_3_) plus a daily dose of 200 mg magnesium, 8 mg zinc, and 400 IU vitamin D for a 3-month period has been found to significantly increase the serum vitamin D level and 6MWD in 22 patients with pulmonary hypertension and vitamin D deficiency. It also led to improvements in right ventricle size and function [81]. While the evidence from this single study is limited, it does show an interesting potential of correcting vitamin D deficiencies to improve the quality of life of PAH patients. Further rigorously conducted randomized trials into the effect of vitamin D supplementation should confirm potential effects on the right ventricle, exercise tolerance, and quality of life.

## Exercise interventions

In the past, exercise training was not recommended in patients with PAH. It was thought that exercise could be a major risk due to increased blood flow, a drop in cardiac output that may worsen right ventricular function or risk of arrhythmia and hypoxia [85–87]. However, more and more studies now show that exercise training can actually be safe and beneficial for exercise capacity, peak oxygen capacity, systemic pulmonary artery pressure, heart rate, and quality of life of patients with various forms of pulmonary hypertension [88–92].

There are different kinds of exercise training that are used in cardiac and pulmonary diseases: (1) aerobic exercise training, like cycling or walking; (2) strength training; and (3) respiratory exercise training [88]. Respiratory training often consists of body perception, yoga, and respiratory muscle strengthening exercises [85]. Although yoga and meditative breathing have been mentioned as possible interventions, studies exclusively examining these interventions were so small that no conclusions can be drawn from them [93].

Studies on exercise training in PAH patients were almost exclusively done in medically stable patients. González-Saiz and colleagues found that aerobic, inspiratory, and muscle resistance training during a short intervention period of 8 weeks can improve muscle power and strength in PAH patients. Also, peak VO_2_ was improved by this exercise intervention [94].

Others found that the combination of aerobic exercise and education can improve fatigue and daily activity, while this effect was not found when giving education only [95].

Different reviews have been published that summarize the effect of exercise interventions combining aerobic exercise, resistance exercise, and/or respiratory training during periods of 3–15 weeks. Sessions were often performed between 2 and 5 times per week. Among others, improvements have been found in 6MWD, fatigue, peak VO_2_, QoL, increased amount of capillaries per muscle fiber, mean pulmonary blood volume, lower resting heart rate, and increased strength of different muscles (including the respiratory muscles) [86–88, 96–98].

Researchers also reported mild exercise-related adverse events, like dizziness, desaturation, progressive fatigue towards the end of the training period, (pre-)syncope, and supraventricular tachycardia in about 5% of the participants [96, 97]. It has been found that fainting during exercise could be triggered in PAH patients when doing isometric exercise training. Therefore, these authors recommended not to imply isometric exercise in training schedules [87].

According to the Pulmonary Hypertension Association (PHA), PAH patients should seek advice regarding safe exercise training from their doctor before starting to exercise. The PHA recommendations for exercise in patients with PAH state that patients should not overexercise to the point of dizziness, chest pain, or severe shortness of breath. Recommended forms of exercise are light resistance training of small muscle groups (without heavy lifting) and light to moderate aerobic activity, such as walking or swimming. For symptomatic PAH patients, it is not advised to exercise the upper and lower body at the same time. Patients with severe exercise intolerance or those with a history of fainting or dizziness are not advised to continue or start a regular exercise program. Lastly, exercise is better avoided during extreme weather circumstances such as very hot or cold temperatures [99].

## Discussion and conclusion

**Fig. 1:**
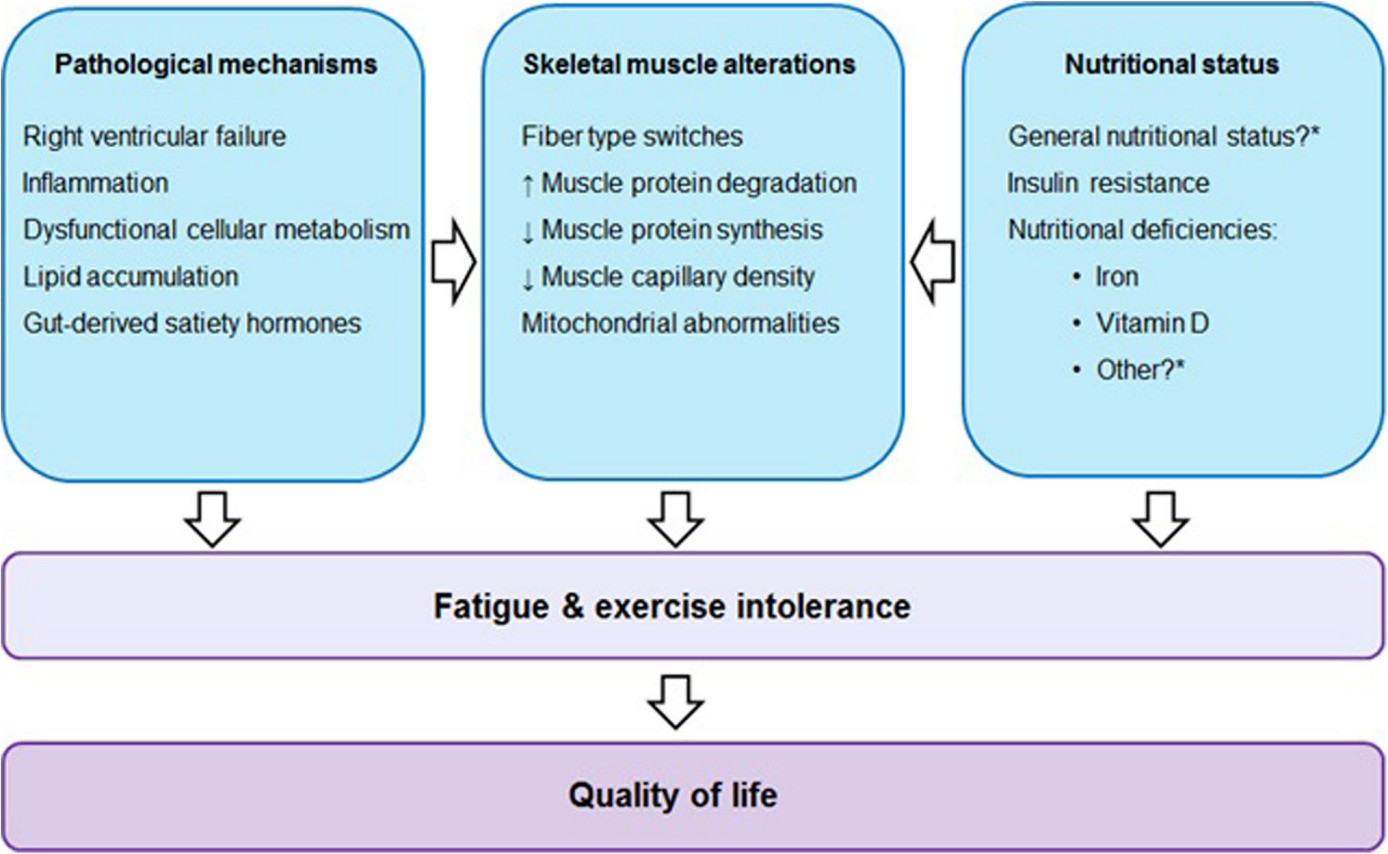
Factors influencing skeletal muscle alterations, fatigue, exercise intolerance, and quality of life in pulmonary arterial hypertension (asterisk, current knowledge gaps)

Nutritional status is likely to be impaired in patients with PAH due to an increased energetic demand, increased protein catabolism, and congestion of the splanchnic organs [2, 3, 17]. The effect and potential side effects of exercise interventions in PAH patients are relatively well studied. Although the pathophysiology of PAH involves many mechanisms that may influence nutritional status and may induce muscle wasting (see Fig. 1), potentially limiting the effect of exercise interventions, there is only little scientific knowledge about the nutritional status of PAH patients and the way this may impact physical activity.

We found no data on the general nutritional status and only limited data on the daily activity of PAH patients. Although deficiencies in micronutrients such as vitamin D, vitamin B_12_, iron, or magnesium might influence symptoms of fatigue and quality of life, there are only few studies into the prevalence of such nutritional deficiencies in PAH patients. Next to micronutrient deficiencies, the presence of insulin resistance is a sign of metabolic change and can also contribute to feelings of fatigue. All of these nutritional deficiencies and metabolic changes can be monitored and treated.

Although it is being recognized that oral iron supplementation in iron-deficient PAH patients is ineffective, there is limited insight in alternative treatments. There are only few studies on the effect of intravenous iron substitution in PAH patients to reduce iron deficiency. Current studies are relatively small with a sample size of 15–20 subjects. Data of a larger study that is currently being performed have not been published yet. Studies on the effect of micronutrients such as vitamin D supplementation in PAH patients are rare and small in size, and study designs can be improved. Chronic inflammation is an important feature in the pathophysiology of PAH, but the effect of nutritional intervention on the inflammation status of these patients is unknown. High-quality papers on the effect of treatments to reduce adverse effects of medication that impact nutritional status are lacking. It should also not be forgotten that physical inactivity itself induces skeletal muscle atrophy [100], so prevention of becoming inactive in the first place should receive extra clinical attention.

In conclusion, larger and well-designed studies into the nutritional status of PAH patients, the prevalence of micronutrient deficiencies, and the effect of supplementation strategies to reduce these deficiencies and improve the quality of life of PAH patients are needed.
